# Sleep and circadian rhythm disruptions in animal models of temporal lobe epilepsy

**DOI:** 10.3389/fnins.2026.1824376

**Published:** 2026-05-08

**Authors:** Sadegh Rahimi, Francesca Silvagni, Pawel Matulewicz, Svenja L. Kreis, Thomas Fenzl, Meinrad Drexel

**Affiliations:** 1 Institute of Pharmacology, Medical University of Innsbruck, Innsbruck, Austria; 2 Department of Anesthesiology and Intensive Care, Technical University of Munich School of Medicine and Health, Munich, Germany

**Keywords:** circadian changes, mouse model, sleep, temporal lobe epilepsy, TLE

## Abstract

Temporal lobe epilepsy (TLE) is frequently accompanied by disruptions to sleep and circadian rhythms, which substantially contribute to disease burden. Human studies are often confounded by antiseizure medications, limiting insight into underlying mechanisms. Animal models therefore provide critical opportunities to examine causal interactions, yet their translational validity has not been systematically evaluated. In this review, we first outline the relevance of rodent models for studying epilepsy- and sleep-related processes. We then examine current evidence for sleep and circadian disturbances across three commonly used TLE models: the pilocarpine (PILO) model, the kainic acid (KA) model, and the traumatic brain injury (TBI) model. We summarize circadian patterns of seizure occurrence, alterations in sleep–wake architecture, and changes in core circadian clock gene expression, as well as alterations in subcortical brain regions involved in sleep–wake regulation. Across models, sleep is consistently fragmented, and circadian molecular machinery is profoundly disrupted, although the direction and magnitude of changes vary by species, protocol, and epilepsy stage. By comparing findings across animal models and patient studies, this review highlights convergences, discrepancies, and key research gaps. Despite variability, animal models remain indispensable for probing the bidirectional links between epilepsy and sleep–circadian regulation.

## 1 Introduction

Temporal Lobe Epilepsy (TLE) is the most common form of focal epilepsy (Téllez-Zenteno and Hernández-Ronquillo, 2012), characterized by recurrent seizures originating in the hippocampus and surrounding mesial temporal structures (Martinez and Peplow, 2023). TLE is often drug-resistant, especially with hippocampal sclerosis, where most patients remain uncontrolled despite treatment (Asadi-Pooya et al., 2017). Beyond seizures, TLE is linked to cognitive impairments, psychiatric disorders such as anxiety (Wei et al., 2023) and depression (Ierusalimsky et al., 2024), and sleep disturbances (Bender et al., 2023). These non-seizure comorbidities significantly contribute to disease burden, yet their underlying mechanisms remain poorly understood.

More than 60% of TLE patients exhibit disrupted sleep (Schiller et al., 2023; Giorelli et al., 2013), underscoring the clinical relevance of this common comorbidity. Sleep disturbances in TLE include subjective complaints such as daytime sleepiness and frequent awakenings (Giorelli et al., 2013), as well as objective alterations, including shortened, fragmented sleep, increased non-rapid eye movement sleep (NREM sleep) stage 1, and reduced rapid eye movement sleep (REM sleep) (Scarlatelli-Lima et al., 2016; Yaranagula et al., 2021; Romigi et al., 2022), as well as altered oscillatory activity during sleep (Schiller et al., 2023; Audrain et al., 2024). Given both their high prevalence and multifaceted nature, these disturbances necessitate a multimodal management strategy. First line interventions include psychoeducation and treatment of comorbid sleep disorders such as obstructive sleep apnea (Nobili et al., 2021). A key therapeutic component is seizure control using anti-seizure medications with minimal sedative or sleep-disrupting effects. Accordingly, agents with relatively favourable sleep profiles, including lacosamide, perampanel, and eslicarbazepine, are often preferred in patients with comorbid sleep complaints (Lupo et al., 2023; Romigi et al., 2020; Liguori et al., 2021). In addition, emerging evidence supports chronobiological interventions and orexin receptor antagonists as potential adjunctive treatments (Berteotti et al., 2023). Despite the high prevalence and clinical impact of sleep disturbances in TLE, consensus on their optimal management remains lacking.

Human studies, while invaluable for establishing the clinical associations between epilepsy, sleep, and circadian rhythms, face inherent limitations that restrict mechanistic investigations. Patient variability in epilepsy type, severity, and response to treatment, coupled with the confounding effects of anti-seizure medications, makes it challenging to isolate the direct impact of epileptic activity on sleep–wake cycles and the underlying circadian system. Furthermore, longitudinal studies from disease onset through chronic progression are often infeasible in humans. Animal models, conversely, allow for controlled experimental environments, the precise induction of epilepsy with known onset and progression, and the manipulation of neuronal circuits to dissect the mechanistic underpinnings of these interactions at cellular, network, and systemic levels.

Although the relationship between sleep and epilepsy has been reviewed extensively in human studies, much less is known about how sleep–wake patterns are affected in animal models of TLE. This represents an important gap, as these models form the basis of much preclinical research and are often assumed to capture key aspects of the human condition. Because this topic has not been systematically reviewed elsewhere, we begin by outlining the relevance of rodent models for studying epilepsy- and sleep-related processes, followed by an examination of current evidence on sleep and circadian disruptions across three commonly used models of TLE. For each model, we summarise circadian patterns of seizure occurrence, alterations in sleep–wake architecture, and changes in the expression of core circadian clock genes. We also review alterations in subcortical brain regions involved in sleep–wake regulation, which may help clarify how these models influence sleep-related processes. Finally, we compare findings from animal studies with available human data, highlight key differences and limitations, and outline priorities for future research.

### 1.1 Pathophysiology of TLE in humans and animal models

TLE in humans is characterised by profound circuit-level reorganisation within mesial temporal structures, most prominently the hippocampus, amygdala, and entorhinal cortex (Blümcke et al., 2013). One of the hallmark pathological features is hippocampal sclerosis, which includes selective neuronal loss in CA1, CA3, and the hilus of the dentate gyrus, accompanied by reactive gliosis (Blümcke et al., 2013; Giachetti et al., 2022). At the circuit level, aberrant connectivity emerges, most notably through mossy fiber sprouting from dentate granule cells, leading to recurrent excitatory loops within the dentate gyrus and CA3 (Puhahn-Schmeiser et al., 2021). In parallel, altered communication between the hippocampus and extra-hippocampal regions, such as the amygdala and parahippocampal cortices (Strýček et al., 2024), contributes to seizure generation, propagation, and the behavioural comorbidities frequently observed in TLE.

Multiple rodent models have been developed to recapitulate TLE pathophysiology, including chemoconvulsant models (pilocarpine, kainic acid), traumatic brain injury (TBI) paradigms, electrical kindling, and genetic models (Jiji et al., 2025). Among these, the pilocarpine (PILO), kainic acid (KA), and TBI models are the most widely used for studying acquired epileptogenesis and chronic epilepsy. Interestingly, all three capture the changes observed in human resected samples from TLE patients.

PILO systemic injection results in a pathology that closely mirrors key features of human TLE, primarily affecting limbic structures such as the hippocampus, amygdala, and thalamus (Choy et al., 2010; Scholl et al., 2013). Pathological hallmarks include significant neuronal loss, particularly in hippocampal CA1 and CA3 regions and the hilus of the dentate gyrus, reactive gliosis, and aberrant synaptic reorganization, most notably mossy fiber sprouting in the dentate gyrus (Huang and Houser, 2024; Junghans et al., 2024; Kyriatzis et al., 2024; Postnikova et al., 2024; Su et al., 2025). These structural changes contribute to the development of chronic hyperexcitability characteristic of TLE. KA injection induces excitotoxicity when administered systemically (Drexel et al., 2011; Drexel et al., 2012; van Nieuwenhuyse et al., 2015) or locally via direct injection into the hippocampus (Rahimi et al., 2023a) or amygdala (Mamad et al., 2023). The resulting neuropathology often mirrors that seen in human TLE, including significant neuronal loss [particularly in hippocampal CA1 and CA3 regions (Hashemi et al., 2023)], gliosis (Drexel et al., 2012), neuroinflammation, and aberrant synaptic reorganization like mossy fiber sprouting (van Nieuwenhuyse et al., 2015), collectively resembling hippocampal sclerosis. TBI injury typically affects cortical regions targeted by trauma (often sensorimotor or parietal), but also the hippocampus (Konduru et al., 2022), and the thalamus (Mroz et al., 2024; Drexel et al., 2015), leading to pathologies such as excitotoxicity, neuroinflammation, neuronal loss, oxidative stress, and aberrant neuronal plasticity (Prakash et al., 2023; Leonard et al., 2024; Chen et al., 2023).

### 1.2 Sleep–wake circuits and circadian regulation in humans and rodents

In rodents, wakefulness is initiated and maintained through the dorsal and ventral ascending arousal systems, a complex neuronal network extending from the brainstem through the thalamus and hypothalamus to cortical areas (Krone et al., 2017; Munn et al., 2021; Jones, 2020). Key neurotransmitters within this network include norepinephrine, serotonin, acetylcholine, histamine, and dopamine (Munn et al., 2021; Jones, 2020). Orexin, originating from the lateral hypothalamus, provides an excitatory drive that sustains activity of the arousal system during wakefulness (Leonard and Ishibashi, 2015; Jones, 2020).

NREM sleep is primarily generated through coordinated activity between thalamocortical networks and sleep-promoting regions in the hypothalamus and brainstem. In particular, GABAergic neurons in the ventrolateral and median preoptic areas (VLPO) inhibit ascending arousal systems, facilitating the transition to and maintenance of NREM sleep (Jones, 2020). Thalamocortical circuits further shape the characteristic oscillatory activity of NREM sleep, including slow waves and sleep spindles (Fernandez and Lüthi, 2020).

REM sleep, although still representing an elusive component of sleep, is primarily generated by a core REM sleep generator located within the mesopontine junction and the pons of the brainstem, which controls initiation, maintenance, REM-specific autonomic changes, and muscle atonia (Luppi et al., 2025; Fraigne et al., 2015; Luppi et al., 2024; Héricé et al., 2019). In addition to the brainstem, structures such as the sublaterodorsal tegmental nucleus, the periaqueductal gray, and parts of the reticular formation contribute to REM sleep regulation (Jones, 2020; Luppi et al., 2025; Luppi et al., 2024). More recently, an excitatory population of corticotropin-releasing hormone–expressing neurons in the dorsomedial medulla has been shown to regulate REM sleep (Schott et al., 2023). Accumulating evidence also suggests that limbic structures, including the claustrum, submammillary nucleus, and basolateral amygdala, contribute to REM sleep regulation and behaviour (Luppi et al., 2025; Luppi et al., 2024). Sleep–wake circuits show remarkable conservation across mammals, with similar brain regions and neurotransmitter systems controlling wakefulness, NREM sleep, and REM sleep in both humans and rodents. The hypothalamus, particularly orexin/hypocretin-producing neurons, serves as a central wake-promoting hub in both species: human fMRI studies reveal posterior and lateral hypothalamic connectivity associated with wakefulness (Boes et al., 2018), while CSF studies in narcoleptic patients demonstrate orexin deficiency linked to excessive daytime sleepiness (Baumann et al., 2006), and rodent chemogenetic activation of lateral hypothalamic glutamatergic neurons causally induce and sustain wakefulness (Wang et al., 2021), establishing clear conservation of hypothalamic wake-promoting function. For NREM sleep, human EEG-fMRI studies reveal that the anterior hypothalamus, including the preoptic area, shows network reorganization and connectivity changes from wake to NREM sleep consistent with a sleep-promoting role (Kaufmann et al., 2006; Jiang et al., 2021); complementing these human findings, rodent optogenetic and chemogenetic studies demonstrate that activation of VLPO GABAergic and galaninergic neurons causally induces NREM sleep, while VLPO lesions produce severe and persistent insomnia with 50–60% reduction in NREM time (Kroeger et al., 2018; Lu et al., 2000; Kostin et al., 2022). In addition, the median preoptic nucleus (MnPO) neurons effectively track sleep pressure (Machado et al., 2022), establishing the preoptic area as a conserved NREM-promoting center across species. REM sleep regulation is centered in brainstem and pontine regions in both humans and rodents. In humans, pontine lesions are associated with excessive REM sleep (Zeidan et al., 2021), while rodent studies have identified mesopontine GABAergic neurons that suppress REM sleep and pontine glutamatergic populations that promote REM sleep (Chen et al., 2022; Weber et al., 2018), supporting a conserved brainstem organization of REM control.

In both humans and rodents, the core molecular clock is generated by a highly conserved transcriptional–translational feedback loop wherein the transcription factors *Clock* and *BMAL1* drive the expression of the repressor genes Period (*PER*) and Cryptochrome (*CRY*), whose protein products accumulate and subsequently inhibit *Clock*/*BMAL1* activity to close the cycle (Lane et al., 2023; Naveed et al., 2024). These cellular clocks communicate through the suprachiasmatic nucleus (SCN) of the hypothalamus, which serves as the master pacemaker in both species, receiving light input via the retinohypothalamic tract and coordinating peripheral oscillators throughout the body (Hung et al., 2025; Foster et al., 2020). The fundamental similarity between humans and mice lies in this conserved molecular machinery and SCN-mediated regulation, with both species exhibiting circadian rhythms driven by the same core clock genes and similar neuroanatomical organization (Lane et al., 2023). However, critical differences exist: mice are nocturnal with fragmented, polyphasic sleep patterns distributed across their rest period, whereas humans are diurnal with consolidated biphasic sleep (Yetish et al., 2015), primarily due to differences in how the circadian system interfaces with sleep–wake regulatory mechanisms, including differential responses to light, and distinct neural outputs from the SCN that determine whether activity is concentrated during dark (nocturnal) or light (diurnal) phases (Foster et al., 2020).

Preclinical studies in rodents have directly shaped human sleep pharmacology by targeting evolutionarily conserved receptors and neural circuits. For example, dual orexin receptor antagonists have emerged from animal research as effective treatments for chronic insomnia (Kishi et al., 2024), while melatonin has been explored as an adjunctive therapy in epilepsy (Liu et al., 2024), illustrating the translational value of animal models for developing novel therapies in neurological disorders.

## 2 Overview of sleep and circadian rhythm disruptions in animal models of TLE

### 2.1 Pilocarpine model of TLE

#### 2.1.1 Circadian pattern of seizure occurrence

The pilocarpine (PILO) model of epilepsy stands as one of the most widely utilized experimental paradigms for studying TLE (Lévesque et al., 2021). Induction typically involves systemic administration of the muscarinic cholinergic agonist PILO, often following pre-treatment with lithium chloride and/or methylscopolamine to limit peripheral effects (Antmen et al., 2025; Zheng et al., 2023; Jagirdar et al., 2015). PILO triggers a period of status epilepticus (SE), which is stopped after generally 2 h to avoid mortality (Ahmed Juvale and Che Has, 2020). Following the initial PILO-induced SE, animals typically enter a “latent” or “silent” period, characterized by the absence of spontaneous seizures, which can last from days to weeks in rats (Chauvière et al., 2012; Lévesque et al., 2021) and 24–48 h in mice (Mazzuferi et al., 2012; Pitsch et al., 2017). Subsequently, animals transition into the chronic phase, marked by the occurrence of spontaneous recurrent seizures (SRSs). Studies using the rat PILO model have reported a circadian pattern in seizure occurrence, with a higher frequency during the light phase (Arida et al., 1999). In mice, seizure clustering was observed at the transition from the light to dark period, specifically between Zeitgeber Time (ZT) 9 to ZT12 (Pitsch et al., 2017). The result is also confirmed by another study (in mice), in which numbers of SRSs were significantly higher during the light phase, with peak numbers of seizures between ZT8 to ZT9 (Liang et al., 2024). However, there are also reports of no correlation between seizure frequency and circadian rhythm (Bajorat et al., 2011) or slightly higher numbers of SRSs during the dark phase (Matos et al., 2010) in rats. In the PILO model, no studies in mice or rats have systematically compared the distribution of SRSs across vigilance states.

#### 2.1.2 Alteration of sleep–wake architecture

Following PILO-induced SE, rats experience significant disruptions in brain oscillation and sleep architecture during both acute and chronic phases. During SE, the physiological hippocampal theta rhythm is largely replaced by high-voltage, fast EEG activity in a rat model (Curia et al., 2008; Mirjebreili et al., 2025; Fu et al., 2022; Chauvière et al., 2009). Hippocampal theta power remains significantly weaker than baseline levels also during the latent phase (Chauvière et al., 2009). As the rat TLE model transitions into the chronic stage, animals exhibit a persistent increase in delta power alongside a significant reduction in theta power across several brain regions, including the CA1 (Pasquetti et al., 2019). These findings, although largely derived from recordings obtained during wakefulness or without systematic discrimination of vigilance states, consistently indicate marked alterations in brain oscillatory activity. However, the alterations are not limited to the brain oscillations. In the acute phase (24 h post-SE), animals show increased wakefulness with decreased NREM and REM sleep (He et al., 2022). Focal PILO administration to the amygdala also reduces both NREM and REM sleep during subsequent light periods (Yi et al., 2015). In the chronic epileptic phase, Matos et al. (2010) reported that the total duration of active wakefulness was significantly reduced over 24 h compared to non-epileptic rats. REM sleep was also affected, with epileptic rats showing a significant reduction in REM sleep during ZT06 to ZT12. Additionally, these animals exhibited a significant increase in NREM sleep across the 24-h cycle, with an abnormal distribution characterized by elevated NREM sleep during both morning and night periods—contrary to the typical biological rhythm in rodents. Beyond changes in individual phases, the overall architecture of the sleep–wake cycle was severely disrupted (Matos et al., 2010).

#### 2.1.3 Disruption of expression of core circadian clock genes

PILO injection influences the expression and oscillation of core circadian genes such as *Bmal1, Clock, Per1, Per2, Cry1,* and *Cry2*, with the effects varying depending on the specific gene, brain region (hippocampus or SCN), species (mice or rats), and the time elapsed since injection.

Observations regarding *Bmal1* include decreased expression in the mouse hippocampus at 14 and 60 days post-injection (Wu et al., 2021), and reduced oscillation and total mRNA in both the SCN and hippocampus of mice at 10–30 days post-injection (Liang et al., 2024). In contrast, *Bmal1* was found to be upregulated in the rat hippocampus several weeks post-injection (Da Santos et al., 2015; Matos et al., 2018), and showed increased oscillation amplitude in the ventral hippocampus of epileptic mice (Debski et al., 2020). *Clock* generally showed downregulation in the rat hippocampus at various time points (Da Santos et al., 2015; Matos et al., 2018), and decreased total mRNA in the mouse SCN and hippocampus (Liang et al., 2024), though some studies in mice found no significant change at various time points (Wu et al., 2021).

*Per1* was upregulated in the light phase and downregulated in the dark phase in the rat hippocampus (Da Santos et al., 2015), while showing increased oscillation and total mRNA in the mouse SCN and hippocampus (Liang et al., 2024), and upregulation at earlier time points in rats and mice (Matos et al., 2018; Popova et al., 2024). *Per2* largely showed no significant change in the hippocampus of mice and rats across several time points (Matos et al., 2018; Wu et al., 2021), but exhibited increased oscillation and total mRNA in the mouse SCN and hippocampus at 10–30 days post-injection (Liang et al., 2024), and increased oscillation in the ventral hippocampus of epileptic mice (Debski et al., 2020), with one study noting downregulation in the mouse hippocampus at 36 h (Popova et al., 2024). *Cry1* was upregulated in the rat hippocampus and mouse hippocampus at certain time points (Da Santos et al., 2015; Matos et al., 2018; Popova et al., 2024) and showed increased oscillation in the ventral hippocampus of epileptic mice (Debski et al., 2020), but decreased total mRNA in the mouse SCN and hippocampus (Liang et al., 2024) and no significant change in the mouse hippocampus in one study (Wu et al., 2021). *Cry2* was downregulated in the rat hippocampus (Da Santos et al., 2015; Matos et al., 2018), and showed decreased total mRNA in the mouse SCN and hippocampus (Liang et al., 2024). These findings underscore the varied and complex effects of PILO injection on the intricate network of core circadian genes (Table 1).

**Table 1:** Changes in core circadian gene expression in the pilocarpine (PILO) model of TLE.

Gene	Region	Species	Time point	Changes	References
*Bmal1*	Hippocampus	Mice	10–30 days post injection	Decreased normalized oscillation amplitude of mRNA expression and decreased total mRNA expression over 24 h	Liang et al. (2024)
	Hippocampus	Mice	14 and 60 days post injection	Downregulated	Wu et al. (2021)
	Hippocampus	Rats	11 weeks post injection	Upregulated	Matos et al. (2018)
	Hippocampus	Rats	12 weeks post injection	Upregulated in dark phase	Da Santos et al. (2015)
	Hippocampus (ventral)	Mice	Chronic epilepsy (time point not mentioned)	Increased oscillation amplitude	Debski et al. (2020)
	SCN	Mice	10–30 days post injection	Decreased normalized oscillation amplitude of mRNA expression and decreased total mRNA expression over 24 h	Liang et al. (2024)
*Clock*	Hippocampus	Mice	1, 3, 14 and 60 days post injection	No significant change	Wu et al. (2021)
	Hippocampus	Mice	10–30 days post injection	Decreased total mRNA expression over 24 h	Liang et al. (2024)
	Hippocampus	Rats	7 days and 11 weeks post injection	Downregulated	Matos et al. (2018)
	Hippocampus	Rats	12 weeks post injection	Downregulated in light and dark phases	Da Santos et al. (2015)
	SCN	Mice	10–30 days post injection	Decreased total mRNA expression over 24 h	Liang et al. (2024)
*Per1*	Hippocampus	Mice	1 h post injection	Upregulated	Popova et al. (2024)
	Hippocampus	Rats	7 days post injection	Upregulated	Matos et al. (2018)
	Hippocampus	Mice	10–30 days post injection	Increased normalized oscillation amplitude of mRNA expression and increased total mRNA expression over 24 h	Liang et al. (2024)
	Hippocampus	Rats	12 weeks post injection	Upregulated (light phase), downregulated (dark phase)	Da Santos et al. (2015)
	SCN	Mice	10–30 days post injection	Increased normalized oscillation amplitude of mRNA expression and increased total mRNA expression over 24 h	Liang et al. (2024)
*Per2*	Hippocampus	Mice	36 h post injection	Downregulated	Popova et al. (2024)
	Hippocampus	Mice	1, 3, 14 and 60 days post injection	No significant change	Wu et al. (2021)
	Hippocampus	Mice	10–30 days post injection	Increased normalized oscillation amplitude of mRNA expression	Liang et al. (2024)
	Hippocampus	Rats	7 days and 11 weeks post injection	No significant change	Matos et al. (2018)
	Hippocampus (ventral)	Mice	Chronic epilepsy (time point not mentioned)	Increased oscillation amplitude	Debski et al. (2020)
	SCN	Mice	10–30 days post injection	Increased normalized oscillation amplitude of mRNA expression and increased total mRNA expression over 24 h	Liang et al. (2024)
*Cry1*	Hippocampus	Mice	8 h post injection	Upregulated	Popova et al. (2024)
	Hippocampus	Rats	7 days post injection	Upregulated	Matos et al. (2018)
	Hippocampus	Mice	1, 3, 14 and 60 days post injection	No significant change	Wu et al. (2021)
	Hippocampus	Mice	10–30 days post injection	Decreased total mRNA expression over 24 h	Liang et al. (2024)
	Hippocampus	Rats	12 weeks post injection	Upregulated in light phase	Da Santos et al. (2015)
	Hippocampus (ventral)	Mice	Chronic epilepsy (time point not mentioned)	Increased oscillation amplitude	Debski et al. (2020)
	SCN	Mice	10–30 days post injection	Decreased total mRNA expression over 24 h	Liang et al. (2024)
*Cry2*	Hippocampus	Mice	10-30 days post injection	Decreased total mRNA expression over 24 h	Liang et al. (2024)
	Hippocampus	Rats	7 days and 11 weeks post injection	Downregulated	Matos et al. (2018)
	Hippocampus	Rats	12 weeks post injection	Downregulated in light phase	Da Santos et al. (2015)
	SCN	Mice	10–30 days post injection	Decreased total mRNA expression over 24 h	Liang et al. (2024)

### 2.2 Kainic acid model of TLE

#### 2.2.1 Circadian pattern of seizure occurrence

The kainic acid (KA) model is one of the most widely utilized experimental models for TLE (Löscher and White, 2023). KA, a potent cyclic analogue of L-glutamate and an agonist of ionotropic AMPA/KA receptors, triggers SE, characterized by continuous or near-continuous seizure activity, often involving stereotyped behaviors like head nodding, wet dog shakes, and generalized convulsions, accompanied by specific electrographic patterns on EEG (Puttachary et al., 2015; van Nieuwenhuyse et al., 2015; Drexel et al., 2012). After a latent period, which can range from days to weeks, the majority of animals develop SRSs, marking the chronic phase of epilepsy (Rahimi et al., 2023a). SRSs by systematic injection of KA in rats showed a significantly higher frequency during the light phase compared to the dark phase (van Nieuwenhuyse et al., 2015; Raedt et al., 2009). However, some research suggests that seizure occurrence in this model might be more closely linked to periods of inactivity per se, rather than strictly to the light/dark cycle itself (Hellier and Dudek, 1999). In mice (intrahippocampal KA) over the chronic phase, SRSs persist in about 60% of mice without a clear preference for light or dark phases (Xing et al., 2026).

#### 2.2.2 Alteration of sleep–wake architecture

Seizures caused by KA injection can change the sleep–wake pattern significantly. Alfaro-Rodríguez et al. (2009) reported that immediately following systematic KA administration, rats exhibited a complete suppression of sleep, spending 100% of the 10-h recording period awake, with neither NREM or REM sleep observed. On the following day, the proportion of time spent awake decreased to approximately 67%, while NREM sleep increased to about 31% and REM sleep accounted for roughly 2% of the total recording time. However, both NREM sleep and REM sleep remained markedly reduced compared to control proportions. Interestingly, by the second day after injection, the distribution of vigilance states had returned to near-normal levels: wakefulness accounted for about 41% of the time, NREM sleep for 49%, and REM sleep for 10%. These proportions did not differ significantly from those of the control group (Alfaro-Rodríguez et al., 2009), indicating that KA-injected rats did not exhibit the expected rebound increase in sleep following sleep deprivation. In mice, KA (intrahippocampal) induces a robust increase in wakefulness and a significant reduction in both NREM and REM sleep during the acute phase, with seizures primarily occurring within approximately 6 h after KA delivery in the light phase. Over the chronic phase, NREM sleep fragmentation remains significant, and REM sleep reduction is notably present during the light phase at day 28 (Xing et al., 2026). In addition, variations in sleep–wake patterns can significantly affect epileptic activity. Low-voltage fast ictal onsets predominantly occurred during wakefulness or REM sleep, while hypersynchronous ictal onsets, the other main type of ictal onset observed, occurred primarily during NREM sleep or periods of immobility (Bragin et al., 1999).

In addition, analysis of spectral power across different phases of epileptogenesis reveals distinct rhythmic signatures associated with seizure progression and network dysfunction. In mice, SE is characterised by significant increases in delta, theta, and gamma power (Puttachary et al., 2015). The chronic phase is characterised by a reduction of peak delta and theta power and an increase in beta and gamma power in the mice’s hippocampus (Tse et al., 2014; Riban et al., 2002; Dugladze et al., 2007). Notably, these significant changes have not been systematically discriminated according to vigilance states, highlighting the need for further studies.

#### 2.2.3 Disruption of expression of core circadian clock genes

Beyond sleep architecture, the KA model of TLE is associated with disruptions in the underlying circadian timing system (Table 2). This is evidenced by the circadian pattern of SRS occurrence noted earlier (van Nieuwenhuyse et al., 2015) and by alterations in the expression of core circadian clock genes within relevant brain regions and even peripheral tissues. However, studies examining clock gene expression in the KA model have yielded somewhat varied results, potentially reflecting differences in species (rat vs. mouse), KA administration route (systemic vs. intrahippocampal), brain regions analysed, and the time point relative to SE (acute vs. chronic phase).

**Table 2:** Changes in core circadian gene expression in the kainic acid (KA) model of TLE.

Gene	Region	KA model, species	Time point	Change	References
*Bmal1*	Hippocampus	Mouse, Intra-amygdala	1 h post injection	Downregulated	Benvenutti et al. (2024)
	Hippocampus	Mouse, Intrahippocampal	3 h post injection	Downregulated	Rambousek et al. (2020)
	Hippocampus	Mouse, Intra-amygdala	8 h and 24 h post injection	Upregulated	de Diego-Garcia et al. (2023)
	Hippocampus	Mouse, Intra-amygdala	14 days post injection	Downregulated	de Diego-Garcia et al. (2023)
	Hippocampus	Mouse, Intrahippocampal	1, 6, 14, 28 days post injection	No significant change	Rambousek et al. (2020)
	Hippocampus	Rat, systemic i.p. injection	9 weeks post injection	No significant change	Yamakawa et al. (2023)
	Hypothalamus	Rat, systemic i.p. injection	9 weeks post injection	upregulated	Yamakawa et al. (2023)
	Liver	Rat, systemic i.p. injection	9 weeks post injection	Upregulated	Yamakawa et al. (2023)
	Small Intestine	Rat, systemic i.p. injection	9 weeks post injection	No significant change	Yamakawa et al. (2023)
*Clock*	Hippocampus	Mouse, Intra-amygdala	1 day post injection	No significant change	Benvenutti et al. (2024)
	Hippocampus	Mouse, Intrahippocampal	3 h post injection	Downregulated	Rambousek et al. (2020)
	Hippocampus	Mouse, Intra-amygdala	8 h, 24 h mRNA post injection	mRNA levels were increased	de Diego-Garcia et al. (2023)
	Hippocampus	Mouse, Intra-amygdala	14 days post injection	No significant change	de Diego-Garcia et al. (2023)
	Hippocampus	Mouse, Intrahippocampal	1, 6, 14, 28 days post injection	No significant change	Rambousek et al. (2020)
	Hippocampus	Rat, systemic i.p. injection	9 weeks post injection	Upregulated	Yamakawa et al. (2023)
	Hypothalamus	Rat, systemic i.p. injection	9 weeks post injection	Upregulated	Yamakawa et al. (2023)
	Liver	Rat, systemic i.p. injection	9 weeks post injection	Upregulated	Yamakawa et al. (2023)
	Small Intestine	Rat, systemic i.p. injection	9 weeks post injection	No significant change	Yamakawa et al. (2023)
*Per1*	Hippocampus	Mouse, Intra-amygdala	1 day post injection (only at ZT-20 out of several time points)	Downregulated	Benvenutti et al. (2024)
	Hippocampus	Mouse, Intrahippocampal	3 h post injection	Upregulated	Rambousek et al. (2020)
	Hippocampus	Mouse, Intrahippocampal	1, 6, 14, 28 days post injection	No significant change	Rambousek et al. (2020)
	Hippocampus	Rat, systemic i.p. injection	9 weeks post injection	No significant change	Yamakawa et al. (2023)
	Hypothalamus	Rat, systemic i.p. injection	9 weeks post injection	No significant change	Yamakawa et al. (2023)
	Liver	Rat, systemic i.p. injection	9 weeks post injection	Downregulated	Yamakawa et al. (2023)
	Small Intestine	Rat, systemic i.p. injection	9 weeks post injection	No significant change	Yamakawa et al. (2023)
*Per2*	Hippocampus	Mouse, Intrahippocampal	3 h post injection	Upregulated	Rambousek et al. (2020)
	Hippocampus	Mouse, Intra-amygdala KA	8 h and 24 h post injection	Upregulated	de Diego-Garcia et al. (2023)
	Hippocampus	Mouse, Intra-amygdala KA	14 days post injection	No significant change	de Diego-Garcia et al. (2023)
	Hippocampus	Mouse, Intrahippocampal	1, 6, 14, 28 days post injection	No significant change	Rambousek et al. (2020)
*Cry1*	Hippocampus	Mouse, Intrahippocampal	3 h post injection	No significant change	Rambousek et al. (2020)
	Hippocampus	Mouse, Intra-amygdala KA	8 h and 24 h post injection	No significant change	de Diego-Garcia et al. (2023)
	Hippocampus	Mouse, Intra-amygdala	8 h post injection	No significant change	de Diego-Garcia et al. (2023)
	Hippocampus	Mouse, Intra-amygdala	14 days post injection	No significant change	de Diego-Garcia et al. (2023)
	Hippocampus	Mouse, Intra-amygdala KA	14 days post injection	No significant change	de Diego-Garcia et al. (2023)
	Hippocampus	Mouse, Intrahippocampal	1, 6, 14, 28 days post injection	No significant change	Rambousek et al. (2020)
	Hippocampus	Rat, systemic i.p. injection	9 weeks post injection	Upregulated	Yamakawa et al. (2023)
	Hypothalamus	Rat, systemic i.p. injection	9 weeks post injection	Upregulated	Yamakawa et al. (2023)
	Liver	Rat, systemic i.p. injection	9 weeks post injection	Upregulated	Yamakawa et al. (2023)
	Small Intestine	Rat, systemic i.p. injection	9 weeks post injection	No significant change	Yamakawa et al. (2023)
*Cry2*	Hippocampus	Mouse, Intrahippocampal	3 h post injection	No significant change	Rambousek et al. (2020)
	Hippocampus	Mouse, Intrahippocampal	1, 6, 14, 28 day post injection	No significant change	Rambousek et al. (2020)

Studies on *Bmal1* showed diverse responses depending on the experimental setup. For instance, *Bmal1* was downregulated in the mouse hippocampus 1 h post-intra-amygdala KA injection (Benvenutti et al., 2024) and 3 h post-intrahippocampal KA injection (Rambousek et al., 2020). Conversely, it showed no significant change in the hippocampus of mice at later time points (1, 6, 14, and 28 days post-intrahippocampal KA injection) (Rambousek et al., 2020) or in rats with chronic epilepsy after systemic KA injection (Yamakawa et al., 2023). Interestingly, *Bmal1* was upregulated in the hypothalamus and liver of rats with chronic epilepsy (Yamakawa et al., 2023). Furthermore, an upregulation of *Bmal1* was observed in the mouse hippocampus 8 h post-intra-amygdala KA injection, but a downregulation was noted after 14 days in the same animals (de Diego-Garcia et al., 2023). The contrasting changes over time imply that *Bmal1* is dynamically regulated in response to the epileptic insult and might have distinct roles in acute vs. chronic phases.

A downregulation of *Clock* gene was reported in the mouse hippocampus 3 h post-intrahippocampal KA injection (Rambousek et al., 2020), however, this gene exhibited a largely stable expression, with no significant change observed in the mouse hippocampus 1, 6, 14, and 28 days post-intrahippocampal injection (Rambousek et al., 2020). In chronic epilepsy models in rats, *Clock* showed upregulation in the hippocampus, hypothalamus, and liver (Yamakawa et al., 2023). *Clock* mRNA levels were increased in the mouse hippocampus at 8 h and 24 h post-intra-amygdala KA injection, but no significant change was observed at 14 days post-injection (de Diego-Garcia et al., 2023).

Regarding other clock genes, *Per1* was upregulated in the mouse hippocampus 3 h post-intrahippocampal KA injection, with no significant change at later time points in the same model (Rambousek et al., 2020). In rats with chronic epilepsy, *Per1* showed no significant change in the hippocampus and hypothalamus, but was downregulated in the liver (Yamakawa et al., 2023). The expression of *Per2* was increased in mouse intrahippocampal and intraamygdala KA models 3 and 8 h after injection, respectively (Rambousek et al., 2020; de Diego-Garcia et al., 2023). *Cry1* was upregulated in the rat hippocampus, hypothalamus, and liver in chronic epilepsy (Yamakawa et al., 2023), however, *Cry2* showed no significant changes in expression in the hippocampus across various KA injection models and time points (Rambousek et al., 2020).

### 2.3 Traumatic brain injury model of TLE

#### 2.3.1 Circadian pattern of seizure occurrence

Traumatic brain injury (TBI), resulting from an external mechanical force applied to the head, is a significant global health issue, affecting an estimated 70 million individuals annually (Dewan et al., 2019). Post-traumatic epilepsy (PTE), characterized by recurrent, unprovoked seizures following TBI, is a common and often devastating long-term consequence, accounting for a substantial portion of acquired epilepsies, particularly in young adults (Kazis et al., 2024).

The progression from TBI to PTE often involves a latent period that can range weeks to months. Following TBI, almost all mice (Di Sapia et al., 2023) experience acute symptomatic seizures—those caused by the acute injury—within about 3 days following TBI, with the first seizure occurring after 18.4 ± 15.1 h post injury (Andrade et al., 2019). Following injury in rats, approximately 25% developed SRSs within 6 months and 50% within 12 months (Dulla and Pitkänen, 2021). Similarly, after repetitive blast TBI in mice, 67% developed SRSs within 9 months (Vigil et al., 2023). Spontaneous seizures in rats are nearly equally distributed over the light and dark phases (Andrade et al., 2017). However, it is reported that seizures in rats overwhelmingly occur during sleep rather than wakefulness (even in the dark phase), as 92% of spontaneous generalized seizures occur during the transition from NREM to REM sleep (Andrade et al., 2017). Upon TBI in mice, the seizures post-injury and also during PTE happen equally during the wake and sleep periods (Vigil et al., 2023). However, the daily number of spikes and sharp waves recorded from electrodes contralateral to the injury site showed a clear circadian distribution in PTE mice, peaking at the onset of and during the dark phase (Di Sapia et al., 2023).

#### 2.3.2 Alteration of sleep–wake architecture

Acutely following TBI in mice, a hypersomnia phenotype characterized by reduced time spent awake has been observed (Vigil et al., 2023). However, reports more commonly describe insomnia-like features and fragmented sleep patterns. Studies using different TBI models in rodents demonstrate increased sleep fragmentation, evidenced by shorter wake and NREM sleep bout lengths, an increased number of transitions between sleep–wake states, and a higher sleep fragmentation index (SFI), compared to controls (Andrade et al., 2022; Konduru et al., 2021; Fox et al., 2024). REM sleep duration has been reported to be reduced following TBI (Andrade et al., 2022), and mice that developed PTE showed a considerable REM sleep reduction (Vigil et al., 2023). NREM sleep, particularly within the delta band (0.5–4 Hz), showed significant changes. Multiple studies using mice report an increase in NREM delta power both acutely and chronically after TBI compared to non-injured controls (Konduru et al., 2022; Konduru et al., 2021). Furthermore, TBI can disrupt the normal homeostatic diurnal oscillation of NREM delta power, which typically declines across the sleep period and increases across the wake period (Konduru et al., 2022). Intriguingly, mice that developed PTE exhibited *lower* NREM delta power compared to TBI mice that did not develop seizures (Konduru et al., 2021). Sleep spindles, characteristic oscillations of NREM stage 2 (N2) sleep occurring during the transition to REM, were also altered; TBI led to a reduction in spindle duration and dominant spindle oscillation frequency, particularly in animals that developed seizures (Konduru et al., 2021; Andrade et al., 2017).

#### 2.3.3 Disruption of expression of core circadian clock genes

Beyond alterations in sleep architecture, TBI also leads to disruptions in circadian rhythms (Di Sapia et al., 2023), as noted previously. Literature focusing on PTE after TBI focused primarily on phenotypic outcomes and downstream pathological markers [e.g., altered GABAergic inhibition (Konduru et al., 2022), TDP-43 accumulation (Vigil et al., 2023), changes in delta power or SFI (Konduru et al., 2021)] rather than detailing the direct upregulation or downregulation of specific genes in response to TBI in animals who developed epilepsy. However, in the studies, which did not discuss epilepsy and did not record EEG for more than 48 h, profound dysregulation of the core molecular machinery of the circadian clock genes were reported. These molecular changes were not confined to the master clock in the suprachiasmatic nucleus (SCN) but extended to other brain regions, such as the hippocampus, cortex, and cerebellum, and to the liver (Table 3).

**Table 3:** Changes in core circadian gene expression in first 48 h after traumatic brain injury (TBI).

Gene	Region	TBI model, species	Time point	Change	References
*Bmal1*	Hippocampus	Fluid-percussion TBI, rats	44 h post injury	Downregulated	Boone et al. (2012)
	SCN	Fluid-percussion TBI, rats	44 h post injury	Upregulated	Boone et al. (2012)
	Hypothalamus	RmTBI, adolescent rats	Two days after last injury	Altered rhythm / Late-night suppression (male mice only)	Sgro et al. (2023)
	Hypothalamus	CCI TBI, mice	24 h post injury	Dysregulated diurnal expression	Korthas et al. (2022)
	Cortex	CCI TBI, mice	24 h post injury	Dysregulated diurnal expression	Korthas et al. (2022)
	Brainstem	CCI TBI, mice	24 h post injury	Dysregulated diurnal expression	Korthas et al. (2022)
	Cerebellum	RmTBI, adolescent rats	Two days after last injury	Rhythmic expression knocked out	Sgro et al. (2023)
*Clock*	Hypothalamus	CCI TBI, mice	24 h post injury	Dysregulated diurnal expression	Korthas et al. (2022)
	Cortex	CCI TBI, mice	24 h post injury	Dysregulated diurnal expression	Korthas et al. (2022)
	Brainstem	CCI TBI, mice	24 h post injury	Dysregulated diurnal expression	Korthas et al. (2022)
	Cerebellum	RmTBI, adolescent rats	Two days after last injury	Rhythmic expression knocked out	Sgro et al. (2023)
*Per1*	Hippocampus	Multiple mTBIs, mice	Two days after last injury	Upregulated	Sgro et al. (2023)
	Hypothalamus	Multiple mTBIs, mice	Two days after last injury	Upregulated	Sgro et al. (2023)
	Hypothalamus	CCI TBI, mice	24 h post injury	Dysregulated diurnal expression	Korthas et al. (2022)
	Cortex	CCI TBI, mice	24 h post injury	Dysregulated diurnal expression	Korthas et al. (2022)
	Brainstem	CCI TBI, mice	24 h post injury	Dysregulated diurnal expression	Korthas et al. (2022)
	Cerebellum	RmTBI, adolescent rats	Two days after last injury	Rhythmic expression knocked out	Sgro et al. (2023)
*Per2*	Hippocampus	Fluid-percussion TBI, rats	4 h & 32 h post injury	Downregulated	Boone et al. (2012)
	Hypothalamus	CCI TBI, mice	24 h post injury	Dysregulated diurnal expression	Korthas et al. (2022)
	Cortex	CCI TBI, mice	24 h post injury	Dysregulated diurnal expression	Korthas et al. (2022)
	Brainstem	CCI TBI, mice	24 h post injury	Dysregulated diurnal expression	Korthas et al. (2022)
	Cerebellum	RmTBI, adolescent rats	Two days after last injury	Rhythmic expression knocked out	Sgro et al. (2023)
	Liver	RmTBI, adolescent rats	Two days after last injury	Rhythmic expression knocked out	Sgro et al. (2023)
*Cry1*	Hippocampus	Fluid-percussion TBI, rats	20 h & 44 h post injury	Downregulated	Boone et al. (2012)
	SCN	Fluid-percussion TBI, rats	20 h post injury	Upregulated	Boone et al. (2012)
	Hypothalamus	RmTBI, adolescent rats	Two days after last injury	Altered rhythm / Suppressed late night (males)	Sgro et al. (2023)
	Hypothalamus	CCI TBI, mice	24 h post injury	Dysregulated diurnal expression	Korthas et al. (2022)
	Cortex	CCI TBI, mice	24 h post injury	Dysregulated diurnal expression	Korthas et al. (2022)
	Brainstem	CCI TBI, mice	24 h post injury	Dysregulated diurnal expression	Korthas et al. (2022)
	Cerebellum	RmTBI, adolescent rats	Two days after last injury	Rhythmic expression knocked out	Sgro et al. (2023)
	Small Intestine	RmTBI, adolescent rats	Two days after last injury	Dysregulated diurnal expression	Sgro et al. (2023)
*Cry2*	Hypothalamus	CCI TBI, mice	24 h post injury	Dysregulated diurnal expression	Korthas et al. (2022)
	Cortex	CCI TBI, mice	24 h post injury	Dysregulated diurnal expression	Korthas et al. (2022)
	Brainstem	CCI TBI, mice	24 h post injury	Dysregulated diurnal expression	Korthas et al. (2022)

Following TBI, *Bmal1* expression can be increased in the SCN while being reduced in the hippocampus (Boone et al., 2012); its rhythmic expression is often lost or dysregulated in the hypothalamus, cortex, brainstem, and cerebellum (Korthas et al., 2022; Sgro et al., 2023; Mountney et al., 2021). *Per* genes (e.g., *Per1*, *Per2*) exhibit disrupted oscillations or altered expression levels across multiple brain regions; for example, *Per1* can be upregulated in the hypothalamus and hippocampus after multiple rmTBIs (Sgro et al., 2023), while *Per2* expression may be reduced in the hippocampus post TBI (Boone et al., 2012) or its rhythmicity lost in the liver (Sgro et al., 2023). Similarly, *Cry1* expression is significantly affected, showing increased levels in the SCN but reduced levels in the hippocampus (Boone et al., 2012), alongside altered or lost rhythmicity in the hypothalamus, cortex, cerebellum, and small intestine (Korthas et al., 2022; Sgro et al., 2023). Other clock-associated genes like *Rev-erb-α* and *Timeless* also demonstrate TBI-induced changes in their expression patterns in specific brain areas (Sgro et al., 2023; Boone et al., 2012; Sabir et al., 2015), collectively indicating a severe disruption of the transcriptional-translational feedback loops essential for maintaining circadian timing after injury (all studies were conducted in <48 h after injury). These changes can definitely affect PTE, however, further studies focusing on epilepsy after TBI and assessing the expression of circadian genes in PTE mice are needed to confirm this assumption.

## 3 Histological changes in sleep–wake regulatory regions observed in animal models of TLE

Beyond the alterations in circadian gene expression observed in the SCN and brainstem following TBI and PILO models of TLE, both KA and PILO models induce widespread histopathological changes in the subcortical brain regions that are central to sleep–wake regulation. These changes may underlie the prominent sleep disturbances commonly reported in TLE (Table 4).

**Table 4:** Cellular changes in sleep–wake regulatory regions in animal models of temporal lobe epilepsy (TLE).

Brain region (caudal to rostral)	Model used	Observation	References
Preoptic region (Medial Preoptic Nucleus - MPO; Magnocellular Preoptic Nucleus - MCPO)	Rat, PILO	MPO: Silver-stained (injured/dying) cells present 2.5 h post-SE, number reached maximum at 8 h, then diminished.	Covolan and Mello (2000)
	Rat, KA	MPO: Silver-stained (injured/dying) cells observed 2.5 h post-SE, number reached maximum at 8 h, lasting up to 48 h post-SE	Covolan and Mello (2000)
	Rat, KA	MCPO: Significant increase in somatic volume and total cell number of parvalbumin-immunoreactive neurons.	Carvalho et al. (2023)
Lateral hypothalamus	Rat, PILO	Few silver-stained (injured/dying) cells present at only 8 h post-SE	Covolan and Mello (2000)
	Rat, KA	Silver-stained (injured/dying) cells observed 2.5 h post-SE, number reached maximum at 8 h, lasting up to 48 h post-SE	Covolan and Mello (2000)
Median raphe	Rat, KA	Approximately twice as many 5-HT-stained (serotonergic) cells compared to controls.	Maia et al. (2019)
Dorsal raphe	Rat, KA	Approximately 30% fewer 5-HT-stained (serotonergic) cells in the DR compared to controls.	Maia et al. (2019)
Laterodorsal tegmental nucleus	Rat, KA	No change in the total number of VAChT-immunoreactive (cholinergic) cells. Trend towards larger volume of VAChT-IR cells (enhanced physiological activity), but not statistically significant.	Soares et al. (2018)
Pedunculopontine nucleus	Rat, KA	No change in the total number of VAChT-immunoreactive (cholinergic) cells. Approximately 25% larger volume of VAChT-IR cells.	Soares et al. (2018)

The lateral hypothalamus (LH) serves as a critical hub in the regulation of vigilance states, containing distinct neuronal populations involved in both sleep and wake promotion (Hassani et al., 2009). Histological studies have shown that the LH undergoes neuronal injury in both KA and PILO models, with the damage being more pronounced and sustained in the KA model (Covolan and Mello, 2000).

In addition to the LH, the preoptic area of the hypothalamus—particularly the median preoptic nucleus (MnPO)—shows signs of cell death and injury in both models (Covolan and Mello, 2000; Carvalho et al., 2023). This region plays a vital role in sleep initiation and homeostasis (Machado et al., 2022), suggesting that its disruption may contribute to impaired sleep regulation in TLE.

The brainstem’s serotonergic system is also affected. The dorsal raphe nucleus (DRN), whose serotonergic neurons are maximally active during wakefulness (Monti, 2010), plays a central role in arousal; optogenetic stimulation of these neurons can induce wakefulness from both NREM and REM sleep (Wang et al., 2024). In contrast, the median raphe nucleus (MRN) modulates theta activity and contributes to sleep maintenance (Huang et al., 2022). Following KA administration in rats, the MRN exhibited approximately a twofold increase in serotonergic neuron counts, whereas the DRN displayed a 30% reduction compared to controls (Maia et al., 2019), indicating a region-specific serotonergic imbalance that could alter sleep–wake dynamics.

Moreover, cholinergic nuclei within the brainstem, including the pedunculopontine tegmental nucleus (PPT) and laterodorsal tegmental nucleus (LDT), also exhibit morphological alterations in the KA model. While the number of cholinergic neurons remained unchanged, their soma size increased by approximately 25% in the PPT and showed a trend toward enlargement in the LDT, potentially reflecting increased neuronal activity (Soares et al., 2018). Both the LDT and PPT are crucial for promoting arousal and REM sleep, as their cholinergic neurons are most active during these states (Boucetta et al., 2014), and optogenetic activation of these neurons reliably induces REM sleep (van Dort et al., 2015).

It should be emphasized that some key sleep–wake regulatory regions (e.g., locus coeruleus, tuberomammillary nucleus [Rahimi et al., 2024)] have not been systematically investigated in the context of these epilepsy models, highlighting the need for further studies focusing on brain areas beyond the primary seizure foci.

## 4 Discussion and future directions

This review summarizes current knowledge on sleep–wake and circadian rhythm alterations in animal models of TLE. As shown in Table 5, available studies consistently indicate that TLE is associated with marked disruptions of sleep and circadian processes. These observations suggest that dysregulation of sleep-regulatory and circadian mechanisms is a characteristic feature of the epileptic state. However, considerable variability across studies and notable gaps in the literature limit current understanding, particularly when comparing findings with reports from human patients.

**Table 5:** Current knowledge and gaps in sleep–wake and circadian alterations in animal models of TLE.

Animal model of TLE	Circadian rhythm of seizures	Changes in sleep–wake architecture immediately after injury	Changes in sleep–wake architecture in chronic Phase	Changes in core circadian clock genes immediately after injury	Changes in core circadian clock genes in chronic phase	Histological changes in sleep–wake regulatory regions
PILO (Mouse)	Light-phase dominance	Increase in wakefulness; decrease in NREM and REM sleep	No data available	Some changes reported	Extensive changes reported	No data available
PILO (Rat)	No clear dominance for light or dark phase	No data available	Increase in wakefulness; decrease in NREM; REM decrease only between ZT06–ZT12	No data available	Extensive changes reported	Some changes reported
KA (Mouse)	No clear dominance for light or dark phase	Increase in wakefulness; decrease in NREM and REM sleep	Sleep fragmentation on post injection day 21 and 28; significant reduction in REM sleep (only during light phase on post injection day 28)	Some changes reported	Almost no significant changes reported	No data available
KA (Rat)	Light-phase dominance	Significant increase in wakefulness; significant decrease in NREM and REM sleep	No data available	No data available	Some changes reported	Extensive changes reported
TBI (Mouse)	No clear dominance for light or dark phase (distributes equally during sleep and wake)	Significant decrease in wakefulness	Sleep fragmentation; non-significant reduction in REM sleep; oscillatory changes in delta band of NREM sleep	Some changes reported	No data available	No data available
TBI (Rat)	No clear dominance for light or dark phase (occurs exclusively during sleep)	No data available	Sleep fragmentation; non-significant reduction in REM sleep;	Some changes reported	No data available	No data available

**Figure 1:**
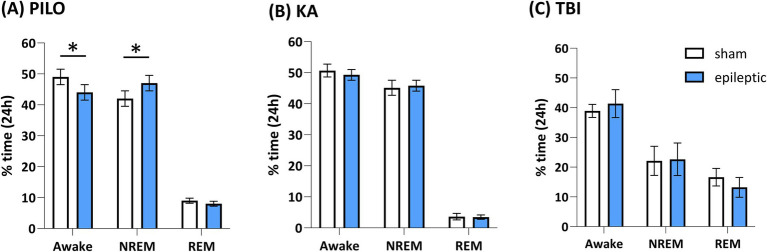
Changes in the percentage of vigilance states in the PILO **(A)**, KA **(B)**, and TBI **(C)** models of TLE over 24-h recordings. Although the relative proportions of different vigilance states are not significantly altered across the models, sleep macroarchitecture is affected in all three conditions (see text for details). **(A)** Adapted from Matos et al. (2010) in rats. **(B)** Adapted from Xing et al. (2026) in mice, obtained from day 21 post-SE. **(C)** Adapted from Andrade et al. (2022) in rats (only animals that developed PTE were included). No other studies have assessed these parameters in mice or rats.

The ultradian and circadian dependency of seizures is well-documented in patients with TLE (Karafin et al., 2010; Gong et al., 2025). A common observation is that seizures occur more frequently during wakefulness, with reported frequencies ranging from 70 to 80% (Ernst et al., 2025; Narang et al., 2022). However, the peak timing of seizures varies considerably across studies. For instance, patients with mesial and mesio-lateral TLE exhibit peak seizure occurrence between 23:00 and 24:00, whereas right TLE patients show a peak between 19:00 and 20:00, and left TLE patients between 19:00 and 22:00 (Gong et al., 2025). Other studies report a peak at 10:00 (Ernst et al., 2025) or a bimodal distribution at 7:00–8:00 and 16:00–17:00 (Karafin et al., 2010). In contrast, animal models present distinct challenges. Chronic seizures following TBI in rats occur almost exclusively during sleep (Andrade et al., 2017) (Figure 1). However, data on seizure timing during sleep vs. wakefulness in PILO and KA models remain limited. Available evidence suggests that in these models, seizures occur predominantly during the light phase, which corresponds to the sleep period in nocturnal rodents (Rahimi et al., 2023b). The heterogeneity in seizure peaks across these models mirrors the variability seen in human TLE, making it difficult to determine which model best recapitulates the human condition.

Human TLE patients often exhibit sleep fragmentation and reduced REM sleep (Scarlatelli-Lima et al., 2016; Yaranagula et al., 2021; Romigi et al., 2022). These features are partially replicated in animal models: PILO, KA and TBI models show consistent sleep fragmentation, and both PILO and TBI rats display significant reductions in REM sleep (Table 5). However, chronic sleep recordings in the KA model are lacking and warrant further investigation. While sleep disturbances in TLE patients are often attributed to anti-epileptic medications (Yeh et al., 2021; Carvalho et al., 2022), similar findings in animal models in absence of medications suggest alternative mechanisms. One possibility is that shared disruptions in core circadian genes contribute to the bidirectional relationship between TLE and sleep–circadian regulation.

**Figure 2:**
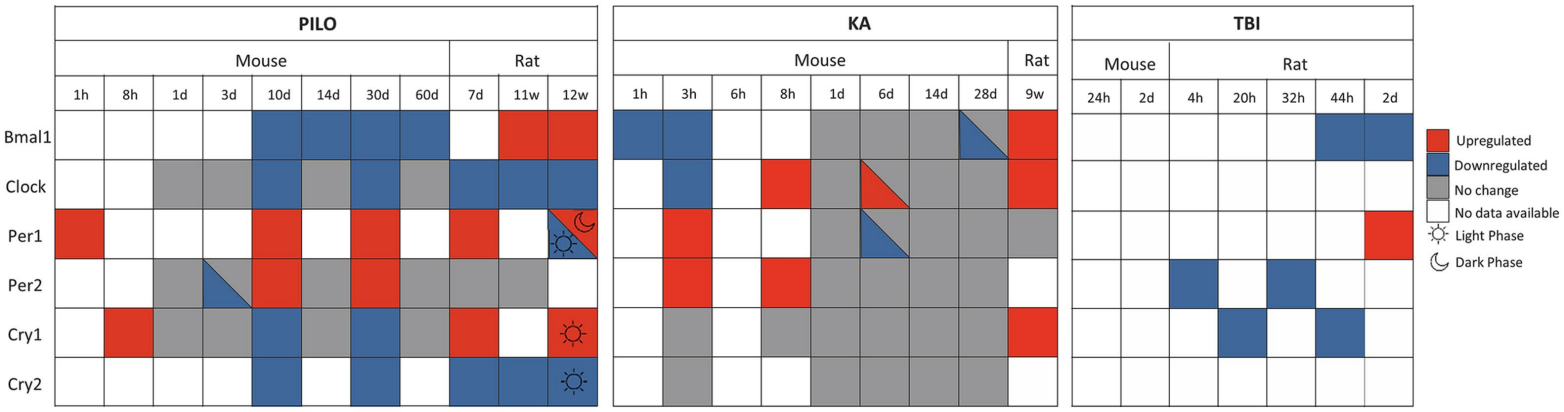
Temporal changes in core circadian clock gene expression in three animal models of TLE. The heatmap shows changes in expression of core circadian genes (Bmal1, Clock, Per1, Per2, Cry1, and Cry2) across different time points following induction of epilepsy or traumatic brain injury in rodent models. The KA (kainic acid) and PILO (pilocarpine) models include time points ranging from acute (1 h) to chronic (2 months) phases, while TBI (traumatic brain injury) data are only available at selected time points between 4 h and 48 h post-injury. Half-colored boxes represent conflicting findings reported by different studies at the same time point, for the same gene and species. References for each data point are listed in Tables 1–3.

In human TLE, only *Bmal1* has been studied in depth. Wu et al. (2021) reported decreased *Bmal1* levels during latent and chronic phases in TLE patients, a similar finding as in the PILO model (mice, chronic phase). However, as illustrated in Figure 2, significant heterogeneity exists across animal models. Notably, the long-term effects of TBI on circadian gene expression remain poorly characterized, as the acute post-TBI alterations in core circadian genes may primarily reflect transient inflammatory responses rather than mechanisms specific to TLE. In contrast, chronic phases of PILO (mice and rats) and KA (rat) models demonstrate widespread dysregulation of core circadian clock genes, implicating these pathways in the pathophysiology of TLE. Despite their potential as therapeutic targets, these genes remain poorly studied in human TLE. Future research should investigate whether analogous gene expression changes also occur in resected brain tissue from patients with pharmacoresistant TLE.

All three animal models of TLE exhibit significant alterations in low-frequency oscillations, although they differ in their temporal progression and qualitative characteristics. Notably, a comprehensive characterization of electrophysiological changes across distinct sleep stages in TLE models remains limited, as most studies have focused on wakefulness, leaving an important gap in our understanding of how sleep architecture is both influenced by and contributes to epileptogenesis. However, the observed changes in delta and theta activity across all three models are not unexpected, given that both the hippocampus and thalamus—structures consistently affected in these models—play central roles in the generation and modulation of theta rhythms and slow-wave sleep (Fernandez and Lüthi, 2020; López-Madrona et al., 2020). In contrast, the sleep fragmentation observed in both human patients and all three animal models is more likely driven by alterations in subcortical regions.

We propose that cellular-level alterations within key subcortical sleep–wake regulatory regions may underlie the changes in sleep macrostructure observed across TLE animal models. Literature review indicates that PILO and KA can significantly affect regions such as the LH, MnPO, and DRN, which are central to bottom-up regulation of vigilance state transitions. Beyond cellular alterations, functional changes have been observed, as reported by altered c-Fos expression in hypothalamic regions following SE caused by PILO in rats (He et al., 2022) and after KA administration in mice (Xing et al., 2026). However, some critical sleep–wake regulatory regions, including the locus coeruleus and tuberomammillary nucleus, remain largely unexplored in these epilepsy models, underscoring the need to extend investigations beyond primary seizure network. In addition, no studies have examined these regions in brain tissue from TLE patients, representing a potentially important future research direction.

Overall, sleep and circadian rhythm alterations in animal models of TLE show high variability, arising from differences in species (mice vs. rats), experimental protocols, and model-specific mechanisms. Nevertheless, there is consistency in the development of chronic seizures accompanied by changes in sleep–wake architecture, alterations in core clock genes, and effects on sleep–wake regulatory regions. Sleep, epilepsy, and circadian gene expression appear to be bidirectionally linked. Core clock genes (e.g., BMAL1, CLOCK, or PER1/2) regulate neuronal excitability, seizure thresholds, and sleep–wake patterns (Jin et al., 2020; Kwon et al., 2025; Re et al., 2020; Gerstner et al., 2014). In turn, as reviewed here, these genes are themselves altered across different animal models of epilepsy. Furthermore, sleep disruption may act as a key mediator. In non-epileptic models, sleep fragmentation is known to alter the amplitude of clock gene oscillations (Rodrigues et al., 2023). Therefore, seizures may exert a dual effect: directly altering gene transcription while indirectly disrupting *the molecular clock through fragmentation of the sleep–wake cycles necessary for its stabilization.*

**Figure 3:**
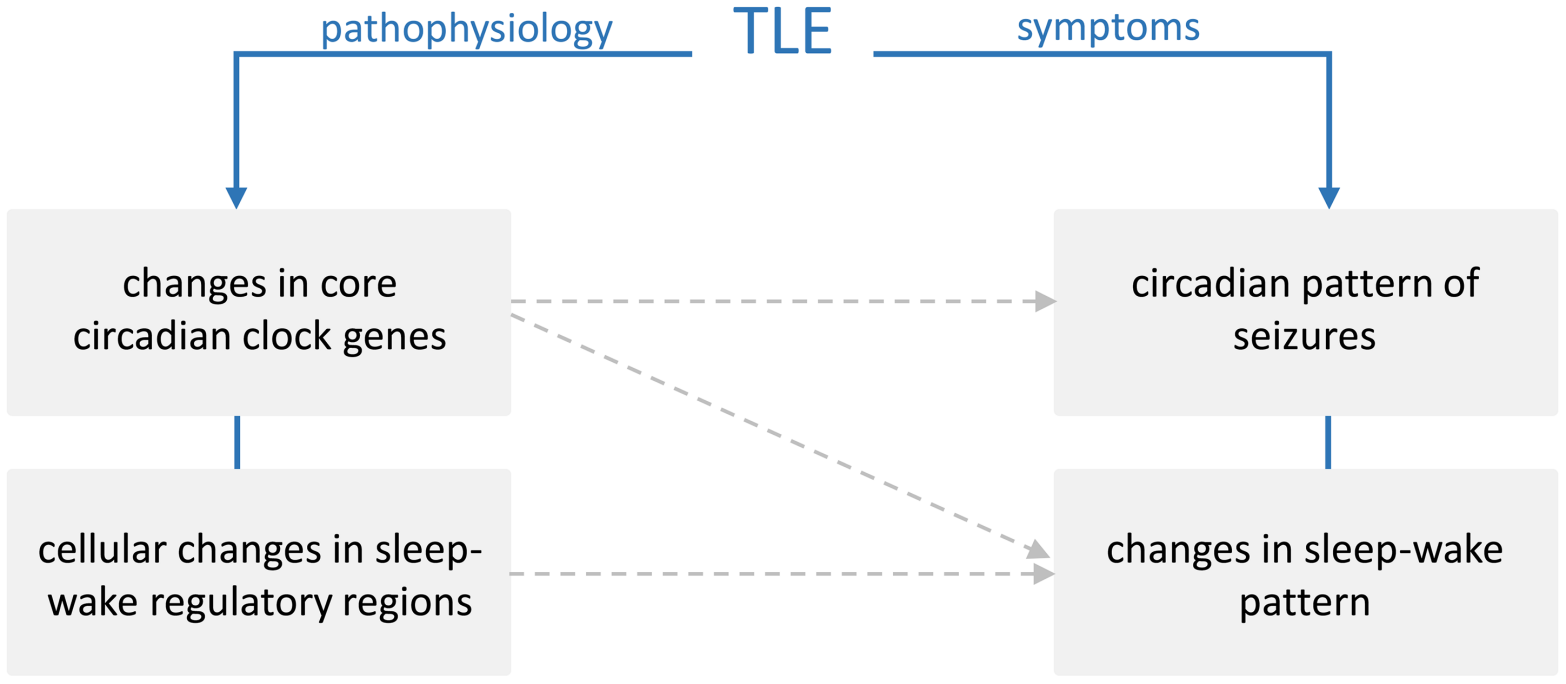
Schematic representation of proposed links between temporal lobe epilepsy (TLE) pathophysiology and symptoms. Changes in core circadian clock genes and cellular alterations in sleep–wake regulatory regions may contribute to the circadian pattern of seizures and disturbances in sleep–wake cycles. Dashed arrows indicate potential effects.

The precise mechanisms linking TLE and sleep-circadian disruptions remain unclear, with many unresolved questions (Figure 3). Given the heterogeneity observed in both patients and animal models, no single model can currently be considered the most valid for directly translating to the human condition. However, the available models remain promising tools for addressing key questions regarding the bidirectional relationship between TLE and sleep–circadian regulation.
